# The State of the Streets

**Published:** 1896-07-18

**Authors:** 


### THE STATE OF THE STREETS.

Every year as the hot weather comes round indignant growls are heard at the evil odours which arise from the London streets, and people are found so ungrateful for the benefits of modern pavements as to attribute to wood or asphalte the smells and irritating dust from which they suffer instead of to lack of care in the cleaning of the streets. It would, however, be foolish to ignore the great sanitary advantages which have accrued from the gradual substitution of impervious pavements for the soft, absorbent, and uneven surfaces with which London was, and to a large extent still is, paved. In the courts and alleys the substitution of asphalte for the old combination of mud and stones has made cleansing a possibility which before was not the case. But where traffic is heavy the substitution of wood for macadam has not always been attended with the same apparent advantages, and there can be no doubt that in many instances the odours and the irritating character of the dust are worse than they used to be with the old style of pavement. This, however, is due to defective cleaning rather than to anything wrong in the wood surface itself. The fact is that the very thing that makes wood an economy in the long run is the cause of its offensiveness. As the traffic rolls over it macadam quickly wears away, the animal excreta deposited on its surface become mixed with the *débris* arising from its own destruction, and the dust or mud so produced is comparatively easily removed. In fact, it is so copious that it must be removed to make the condition at all tolerable.

With wood, however, the wear and tear is comparatively slight, and all the offensiveness deposited upon it lies there, until by great labour it is scraped off, which rarely happens. The vestries have a touching confidence in the variability of our climate, and trust to an occasional thunder shower to soften the adherent cake of manure with which the pavement becomes coated, and when the normal shower fails to come this layer of ordure is left to take its chance. Meanwhile it is pounded by the passing traffic, and scratched by the scrapers of the scavengers, and thus a dust arises, small in quantity no doubt, but of excessive lightness and indescribable offensiveness. The cure, of course, is to wash the streets with water and send all this stuff swirling down the drains.

This is, however, not so simple a matter as it may seem, and is greatly complicated by the fact that the drainage of London is by no means ideal. Everything that goes into the sewers has to be pumped out again, and local sewers are far from always being of modern type, so that although in certain districts the cleansing of the streets is carried on in proper fashion—everything being swept into the sewers with much outpouring of water by hose and jet—in other districts the scavengers may be seen carefully blocking up the gullies and scooping up great pools of sludge to cart away. It is to a large extent the state of the London drains that forces the vestries to use water to "lay the dust" instead of using it to wash the streets. In any case, however, it is the dirt lying on the pavements, not the pavements themselves, which is the cause of their offensiveness.

